# Optimizing Low Crude Protein Diets with Coated Cysteamine Hydrochloride and Exogenous Alkaline Protease Supplementation in Broiler Chickens

**DOI:** 10.3390/vetsci12070622

**Published:** 2025-06-27

**Authors:** Hafiz Abu Bakar Siddique, Ehsaan Ullah Khan, Muhammad Muneeb, Saima Naveed, Elham Assadi Soumeh, Sohail Ahmad, Rashed A. Alhotan, Abdulrahman S. Alharthi, Ala E. Abudabos

**Affiliations:** 1 Department of Animal Nutrition, Faculty of Animal Production and Technology, University of Veterinary and Animal Sciences, Lahore 54000, Pakistan; 2021-mphil-1563@uvas.edu.pk (H.A.B.S.); ehsaan@uvas.edu.pk (E.U.K.); muneebsaqib5826@gmail.com (M.M.); saimamahad@uvas.edu.pk (S.N.); 2 School of Agriculture and Food Sustainability, The University of Queensland, Gatton, QLD 4343, Australia; e.assadisoumeh@uq.edu.au; 3 Department of Poultry Production, Faculty of Animal Production and Technology, University of Veterinary and Animal Sciences, Lahore 54000, Pakistan; 4 Department of Animal Production, College of Food and Agriculture Sciences, King Saud University, P.O. Box 2460, Riyadh 11451, Saudi Arabia; ralhotan@ksu.edu.sa (R.A.A.); abalharthi@ksu.edu.sa (A.S.A.); 5 Department of Agriculture, School of Agriculture and Applied Sciences, Alcorn State University, 1000 ASU Drive, Lorman, MS 39096-7500, USA

**Keywords:** broilers, exogenous protease, crude protein, cysteamine hydrochloride, performance

## Abstract

Decreasing crude protein (CP) in broiler diets can improve sustainability but may compromise growth performance. Feed additives like coated cysteamine hydrochloride (CSH) and exogenous alkaline protease (EAP) can enhance protein utilization and promote gut health. While CSH modulates metabolism, EAP improves digestibility, but their combined effects in low CP diets remain unclear. This study examines the synergistic impact of CSH and EAP on broiler growth, gut histology, carcass traits, immune response, and nutrient digestibility, aiming to optimize performance while reducing environmental impact. Six-hundred, 1-day-old broiler Ross-308 chicks (male) were allotted to four treatments, each consisting of six replicates of twenty-five birds, in a factorial arrangement using a completely randomized design. The treatments comprised two inclusion levels of coated CSH (0.2 and 0.4 g/kg with or without EAP (0 and 0.2 g/kg) in reduced CP diets. A ten percent reduction in CP from the standard requirements of Ross-308 (20.7% vs. 23% in the starter, 19.35% vs. 21.5% in the grower, and 17.55% vs. 19.5% in the finisher phase) was made in all the dietary treatments. A notable interaction (*p* ≤ 0.05) between CSH and EAP was detected in body weight gain (BWG), feed conversion ratio (FCR), carcass characteristics, and gut morphology during the whole study duration. Similarly, nutrient digestibility and immune response were also influenced by the combined use of CSH and EAP. The synergistic use of coated CSH at 0.2 g/kg or 0.4 g/kg with EAP in reduced CP broiler diets can enhance performance, intestinal health, carcass characteristics, immune response, and nutrient digestibility. Implications of these findings in commercial feeding practices could substantially improve the efficiency and sustainability of broiler production systems.

## Simple Summary

Feeding broilers with low crude protein (CP) diets can negatively affect growth performance, but at the same time, it lowers nitrogen excretion to the environment. Feed additives like coated cysteamine hydrochloride (CSH) and exogenous alkaline protease (EAP) improved protein utilization and gut health when fed separately to broilers. These two additives improve metabolism and digestibility, but their combined effects in low CP diets remain untested. This study examines the collaborative impact of CSH and EAP on broiler performance and physiological parameters. In total, 600 one-day-old broiler chicks were divided into four treatments. Two inclusion levels of coated CSH (0.2 and 0.4 g/kg with or without EAP (0 and 0.2 g/kg) were tested in reduced CP diets. A significant interaction between CSH and EAP was detected in body weight gain, feed conversion ratio, carcass characteristics, and gut histology in the study. Nutrient digestibility and immune response were improved when CSH and EAP were combined. It was concluded that the synergistic use of coated CSH with EAP in lower CP broiler diets can enhance broilers’ performance and intestinal health.

## 1. Introduction

Chicken is a common protein source due to its high protein and low-fat content. Industrial poultry farming looks for efficient ways to cut expenses and maximize output; to this end, it is vital to investigate a wider variety of products that can improve broiler production and raise performance metrics. In poultry farming economics, feed costs constitute 65–70%, with 30% derived from protein supply, a critical component affecting chickens’ growth performance [1]. Protein constitutes the costliest and second-largest nutrient in poultry diets. The principal ingredients of chicken feed are maize grains and soybean meal (SBM), which provide energy and protein, respectively [2].

SBM is regarded as the main source of protein in the contemporary poultry production process because of its great digestibility and well-balanced amino acid (AA) profile. SBM is an important commodity, but in addition to its unpredictable supply and seasonal scarcity in some parts of the world, it is also an expensive ingredient [3,4]. Due to the global rise in SBM prices, poultry feed is rising. Accomplishing a certain dietary CP level in feed is essential for broiler growth, and hence, it is a concern for the poultry industry [5]. The recommended CP levels in the Ross-308 diet are 23, 21.5, and 19.50% for the starter, grower, and finisher phases, respectively (Ross-308 Nutrition Specifications, 2022). In response to economic and environmental challenges, a prevailing trend in chicken production is the adoption of low CP diets to mitigate the rising costs of protein ingredients and nitrogen emissions, which are closely associated with CP fermentation by gut microbiota, a critical determinant of chicken growth performance [6]. Excessive dietary CP, an uneven amino acid ratio, the presence of anti-nutritional agents, and insufficient endogenous enzyme availability may all lower the optimum protein digestibility [7].

The small intestine produces and releases many endogenous proteases, which are generally considered sufficient to regulate protein consumption [8,9]. However, early in life, the broiler gastrointestinal tract (GIT) is not supportive enough to efficiently digest certain nutrients [10]. Therefore, considerable amounts (18–20%) of protein move through the gut without complete digestion [11,12]. Thus, a possible solution to enhance protein digestibility in broilers can be the supplementation of exogenous proteases [13,14]. Exogenous proteases are hydrolase enzymes that can cleave the peptide bonds to improve protein digestibility and solubility [15]. In addition, these enzymes may also hydrolyze the proteinaceous anti-nutritional factors [16,17]. Protease supplementation may also assist in lowering the CP content of the broiler diet from 10% to 20% [18,19], provided that the diet is balanced for all the essential AA. As a result, this lowering of CP contents may also contribute to reducing the feed cost [20] and environmental effects. The pH of the GIT varies greatly, from the very acidic proventriculus environment to the comparatively neutral environment of the small intestine [18]. Like other enzymes, proteases are pH-specific, and their effectiveness is impacted by the large pH range of the GIT, which ranges from very acidic (proventriculus) to relatively neutral (small intestine) [18]. The exogenous alkaline protease (EAP) presents a relatively more workable solution in broiler diets [13] since it is more efficient in the neutral to alkaline pH range of the intestines [21], where most of the protein digestion takes place. The EAP acts like endogenous trypsin and chymotrypsin enzymes; trypsin cleaves basic AAs like lysine and arginine, while chymotrypsin cleaves aromatic AAs like tyrosine and phenylalanine [22].

Cysteamine (CS) is a bioactive compound that can be administered orally or intravenously and it has demonstrated the capacity to enhance production performance in various livestock species. CS is also synthetically available as a dietary additive in the form of cysteamine hydrochloride (CSH). It is a stable amino-thiol compound and one of the metabolic enhancers and activity modifiers in living tissue [23]. A neuropeptide called “somatostatin” is extensively present in the digestive tracts of animals. Somatostatin has an inhibitory effect on growth hormones, metabolic hormones, and digestive enzyme secretion from the pancreas and small intestine [23,24] and also an immunosuppressive effect by decreasing the IgA-positive cells in the duodenal and jejunal mucosa [25,26]. Dietary inclusion of CSH exerts an inhibitory effect on somatostatin while increasing the production of growth hormone, which increases the production of insulin-like growth factor, which ultimately may enhance the BWG of broilers [27]. Derivatives of CSH are also reported to promote animal growth by lowering oxidative stress, maintaining intestinal integrity, and enhancing immunity [24,28]. CSH mediated increases in growth hormone secretion and enhances B-cell development, and antibody production from the immune system [29]. Through three distinct mechanisms, CSH functions as an antioxidant: (i) its thiol group acts as an antioxidant; (ii) it raises glutathione (GSH) levels in cells; (iii) it eliminates hydrogen peroxide and can also directly eliminate reactive oxygen species (ROS) and harmful byproducts of lipid peroxidation [30]. The increases in the concentrations of metabolic hormones, thyroxine (T4), and triiodothyronine (T3) have been ascribed to the application of CSH [23,31]. Moreover, the secretions of pancreatic proteases, lipase, and amylase are also attributed to CSH, which leads to improved digestion of proteins, fats, and carbohydrates, respectively.

As previously mentioned, both EAP and CSH complement the activities of endogenous proteases and improve protein utilization in chickens. The beneficial effects of individual supplementation with EAP and CSH in broiler diets are well explained in existing literature. However, no prior studies have explored the possible benefits of the combined application of CSH and EAP supplementation in reduced CP diets of broilers. With increasing pressure to formulate economically viable and environmentally sustainable poultry feeds, strategies that reduce dietary protein content without compromising animal performance are gaining importance. This study was, therefore, planned to determine the impacts of CSH supplementation, with or without EAP, on growth performance, nutrient digestibility, gut histology, immune response, and carcass traits in broiler chickens fed low CP diets. We hypothesized that the synergistic use of CSH and EAP would yield greater benefits than either alone, thereby enhancing broiler performance while reducing the economic burden associated with high-protein diets. This study introduces a novel approach by exploring the interactive effects of CSH and EAP in a low-protein dietary framework—an area that has remained largely unexplored. The findings could have practical implications for the poultry industry, as the optimized use of CSH and EAP might enable producers to formulate cost-effective, low CP diets that maintain or improve broiler productivity. Furthermore, reducing dietary protein levels may contribute to lower nitrogen excretion, thus supporting environmental sustainability and compliance with regulatory standards.

## 2. Materials and Methods

### 2.1. Ethics Statement

The trial was performed at Broiler Experimental Shed, an environmentally controlled, floor-rearing facility at Ravi Campus (A-Block), University of Veterinary and Animal Sciences (UVAS), Lahore, Pakistan, for 35 days. All procedures were accomplished following the guidelines established by the Ethical Review Committee of UVAS, Lahore (Approval No: DR/3/3; Date: 4 July 2023).

### 2.2. Experiment Design and Bird Husbandry

In total, 600 1-day-old male broiler (Ross-308) chicks were randomly divided into four treatments with 6 pens of 25 chicks each. A 2 × 2 factorial design of treatments was implemented under completely randomized design (Table 1). The treatments included (1) basal diet + 0.2 g/kg CSH at diet without protease, (2) basal diet + 0.2 g/kg CSH and 0.2 g/kg EAP 0.2 g/kg, (3) basal diet + 0.4 g/kg CSH without EAP; (4) fed basal diet + 0.4 g/kg CSH and 0.2 g/kg EAP. The CSH used in the current study was supplied by Hangzhou King Techina Technology Co., Ltd. (Hangzhou, China containing 27% CSH). The commercial EAP (CIBENZA DP-100^®^, Novus International, Inc., St. Charles, MO, USA) is a heat-stable, broad-spectrum, light brown powder composed of *Bacillus licheniformis* PWD-1 fermentation soluble combined with ground limestone, exhibiting a minimum enzymatic activity of 600,000 U/g. Both of the additives were in the powdered form and were added to the basal diets during feed processing to prepare the experimental diets.

The chicks were maintained and managed according to the standard management procedures suggested by the Ross-308 [32]. The house temperature was set at 33 °C, which was decreased by 3.0 °C weekly until day 21 and then maintained at 24 °C for the remainder of the study. The litter material used for bedding was rice husk with a layer of 3–4 inches on the floor. During the experimental period, the house was kept under a 23 h light and 1 h dark cycle. Feed and fresh, clean drinking water were supplied ad libitum during the trial. Birds were vaccinated against Newcastle disease virus (NDV) type B1, Lasota strain, and infectious bronchitis (IB) mass-type live virus through individual eye drops on day 1. On day 8, vaccination against the infectious bursal disease virus (IBDV) Gambaro, Cheville 1/68 strain, was administered through drinking water. To boost titers against ND and IB, clone 45 strain of NDV and IB vaccines were repeated at day 20 through drinking water.

The diets were prepared with a 10% decrease in CP from the standard of Ross-308 [33]. However, for the rest of the nutrients, the standards of Ross-308 were followed for the starter (1–10), grower (11–24), and finisher (25–35) phases, respectively (Table 2). Chemical evaluation of feed materials and prepared diets was performed through proximate analysis using the established techniques of the AOAC [34]. All feed samples were ground through a 2 mm screen of Willey Mill (Arthur H. Thomas Co., Vine St & N 3rd St, Philadelphia, PA 19102, USA). For further uniformity of the particles, a 1 mm sieve of cyclone mill (Firistsch^TM^, Pulverisette 14, Frankfurt, Germany) was used. Processed samples were dried in a forced air oven (Model UF260, Memmert GmbH + Co., KG, Schwabach, Germany) for four hours at 105 °C for DM estimation. Crude fiber (CF), crude ash, ether extract (EE), and CP were estimated from dried ground samples. The Micro Kjeldahl Method was used to determine CP content. High-performance liquid chromatography (LC-4500 Compact HPLC System, JASCO International Co., Ltd., Hachioji, Tokyo 192-0046, Japan) was used to analyze the amino acid profile of the feed. The amount of crude ash was estimated using a muffle furnace (Box-Type Resistance Furnace, SX-2.5-10, Top Cloud-Agri Technology Co., Ltd. Hangzhou, China). Crude fiber was estimated using Ankom^®^ fiber analyzer (Ankom Technology, 2052 O’Neil Rd, Macedon, NY 14502, USA). Ankom^®^ fat extractor was used for EE determination.

### 2.3. Sampling and Measurements

The production performance of broilers was assessed to determine the appropriate feed additive strategy. Feed intake (FI), BWG, FCR, and European performance efficiency factors (EPEFs) were measured weekly and computed for the cumulative period [36].

Three birds were randomly chosen from each replicate (n = 18 birds per treatment) for sampling at 35 d for carcass characteristics and blood analysis. Selected birds underwent an 8 h fasting period. The birds were individually weighed, humanely slaughtered, and the carcass components were determined as a percentage of pre-slaughter weight [37].

Blood samples were collected in evacuated tubes for serum separation. The samples were placed for 1 h at room temperature, and then samples were centrifuged at 2000 rpm for 5 min. To test the antibody titers against the Newcastle disease virus (NDV), the collected serum was split into aliquots and then stored at −21 °C [19]. The antibody titer against NDV was determined through hemagglutination-inhibition (HI) and hemagglutination tests as previously described [38].

To assess the small intestinal morphological changes, 2 cm sections from the duodenum (distal to the loop of duodenum) and the ileum (5 cm posterior to the Meckel’s diverticulum) were obtained from three birds per pen (n = 18/treatment). Gentle flushing of luminal contents was performed with Ringer’s lactate solution. A 10% formalin (neutral buffered) solution was used to fix the sample for 48 h before further processing. Dehydration was achieved using altered dilutions of ethyl alcohol, and the samples were embedded in paraffin wax. The paraffin blocks of samples were cut through a precision rotary microtome (CUT 5062, SLEE medical GmbH, Am Neuberg 14 55268 Nieder-Olm, Germany) at 5 μm thickness and stained with eosin (Eosin Y soln, Cat. #EY07-500R, TissuePro Tech. LLC, 2153 SE Hawthorne Road, Gainesville, FL, 32641, USA) and hematoxylin (Hematoxylin soln. Cat. #H08-500R, TissuePro Tech. LLC, USA). The histomorphometric analyses were performed by two independent, skilled observers who were unaware of the trial design. The villus height (VH) and crypt depth (CD) were examined by a light microscope (CX 43, Olympus Co., Ltd., Tokyo, Japan) at 10X objective, and the images were obtained using a color video camera (HD 1000-Lite, Meiji Techno Co., Ltd., 322-1, Chikumazawa, Miyoshi machi, Iruma-gun Saitama 354-0043, Japan). For each section, 10 intact and well-oriented villi were selected for obtaining histomorphological measurements using Pixel Pro^®^ 3.2™ software (Labo America Inc., 920 Auburn CourtFremont, CA 94538, USA).

An indigestible analytical marker to determine the digestibility coefficients of the nutrients [39]. A 1% dietary Celit (10 g/kg diet; Celit^®^ 281, Merk MilliporeSigma KGaA, Darmstadt, Germany) was added to treatments as a digestibility marker from day 32. The excreta samples of birds from each replicate were collected on butter paper after regular intervals of 8 h and were composited for 24 h. After carefully removing the scales, feathers, and feed residue from the excreta trays, the composite excreta were kept in plastic bags in a freezer at −20 °C for further analysis. Moreover, two birds from each replicate (n = 12 per treatment) were used for the collection of pooled ileal digesta on day 35 for ileal digestibility assessment. The ileal contents were promptly placed in sealed bags and stored at −20 °C. Ileal digesta and excreta samples were finely ground and subjected to proximate analysis and AIA after being dried for 72 h at 50 °C in an oven. The nutrient digestibility was assessed as previously described [40].

(1) $$\mathrm{Nutrients digestibilty}\;\left(\%\right)=100-\left(100\times \frac{Mi\times Eo}{Mo\times Ei}\right)$$

where *Mi* is the marker concentration in the diet; *Mo* is the marker concentration in the ileal digesta or excreta; *Eo* is the nutrient concentration in the ileal digesta or excreta; *Ei* is the dietary nutrient concentration.

### 2.4. Statistical Analysis

The data were initially assessed for normality and homogeneity of variance test using the Kolmogorov–Smirnov and Levene’s test, respectively. Subsequently, factorial ANOVA was applied using the PROC GLM in SAS software (Statistical Analysis System, version 9.1), considering CSH and EAP as the main effects, and their interaction was also tested. For performance parameters, the replicate of chickens was considered the experimental unit; for other data, the individual bird was considered the experimental unit. Duncan’s Multiple Range Test was employed to compare means at a significant level of 5%. The results are presented as least square means with pooled SEM.

## 3. Results

### 3.1. Growth Performance

Table 3 depicts the effects of CSH at doses of 0.2 g/kg and 0.4 g/kg of feed with or without EAP in reduced CP diets on broiler performance. The FI exhibited a marked difference, as indicated by the substantial interaction (*p* ≤ 0.05) between CSH and EAP during the experimental period. Similarly, the findings on BWG demonstrated a significant association (*p* < 0.05) between CSH and EAP across the treatments. Correspondingly, the CSH × EAP interaction for the FCR was notable (*p* ≤ 0.05) for the overall experiment duration. The interaction between CSH and EAP was deemed non-significant during the entire trial duration; nonetheless, CSH had a significant effect (*p* ≤ 0.05) on the survival rate of the birds. A notable interaction between CSH and EAP was observed for the EPEF during the overall trial period (*p* ≤ 0.05). The CSH 0.2+ exhibited the highest EPEF relative to all other treatments.

### 3.2. Carcass Characteristics

The results on carcass and organ yields at 35 d are summarized in Table 4. The live body weight varied significantly among the treatments, leading to significant differences in carcass and organ weights. Compared to other dietary treatments, the CSH × EAP interaction for the live body weight, hot carcass, leg quarter, breast, and heart weights was considerably (*p* ≤ 0.05) higher in CSH 0.2+. However, the liver and fat percentages were significantly higher (*p* ≤ 0.05) with CSH 0.4 without EAP. On the other hand, gizzard weight was not influenced by CSH or EAP.

### 3.3. Immune Status

**Figure 1:**
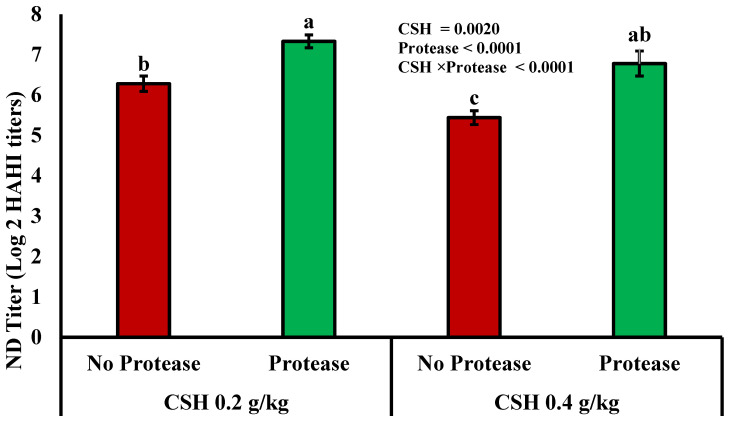
Effects of dietary treatments on the HAHI antibody titers against Newcastle disease (NDV) in broiler chickens at day 35; CSH 0.2 g/kg = cysteamine hydrochloride at 0.2 g/kg; CSH 0.4 g/kg = cysteamine hydrochloride in the diet at 0.4 g/kg; protease was added to the diet at 0.2 g/kg. Superscripts on different bars indicate statistical significance at *p* ≤ 0.05.

The CSH × EAP interaction for the antibody titer against NDV and bursa weight was significant (*p* ≤ 0.05), as shown in Figure 1. The results for CSH 0.2 + EAP were considerably elevated (*p* ≤ 0.05) compared to all other treatments. However, the interaction between CSH and EAP for spleen weight was significant among all the groups (*p* > 0.05). It was also observed that bursa weight was significantly affected by CSH 0.2 vs. CSH 0.4, while EAP did not cause any significant difference (Table 5).

### 3.4. Gut Morphology

The impact of CSH at doses with and without EAP on ileal and duodenal VH, CD, and VH: CD is depicted in Table 6. A substantial interaction was seen between CSH and EAP regarding VH and VH: CD in both segments of the small intestine (*p* < 0.05). The results revealed that CSH 0.2+ has the highest values for histomorphology traits, while CSH 0.4-ve has the lowest values. It was also observed that VH and VH: CD were significantly affected by EAP, but CSH did not show significance (*p* > 0.05). The CD was not affected by any treatment.

### 3.5. Nutrient Digestibility

The CSH × EAP interaction for DM, CP, and OM was significant (*p* ≤ 0.05) in total tract digestibility. The results revealed the highest digestibility of DM, CP, and OM in the CSH 0.2+ group and the lowest in the CSH 0.4 without EAP group (*p* ≤ 0.05). The same trend for the CSH × EAP interaction was found in the ileal digestibility of nutrients (Table 7).

## 4. Discussion

The results revealed that FI was higher in CSH 0.2+EAP treatment and lower in CSH 0.4 without EAP treatment. Our findings align with a previous study [41], which reported a significant increase in the FI of broilers supplemented with CSH. In the literature, it is also evident that higher doses of CSH may lead to low FI [42], which also happened here in CSH 0.4 without EAP. This low FI with a high dose of CSH may be due to the inhibitory effect of higher doses of CSH on the digestive enzymes, regulation of gut hormone levels, and appetite regulation. Furthermore, elevated doses of CSH may lead to stomach ulcers and damage to the gut lining, thereby explaining the reduced feed intake [23]. However, adding exogenous protease ameliorated this effect, and a relatively higher FI was observed in the CSH 0.4+EAP. Contrary to our study, Yang et al. [23] found a non-significant impact of CSH on the FI of broilers. CSH × EAP interaction was found significant in BWG of broilers in the overall trial period of 35 days. The result of this study agreed with those of [23], who found that CSH at 9 mg/kg showed the highest BWG, and at 150 mg/kg and 200 mg/kg of feed had the lowest BWG. Similarly, Zavy and Lindsey [41] also found that CSH administered at 1200 mg/kg feed decreased BWG in broilers. In contrast to our findings, Nunes et al. [42] found no statistical significant improvement in the BWG of broilers with CSH. The increase in the BWG of broilers with CSH might be due to the inhibition of somatostatin and the resulting increased level of growth hormone. Moreover, CSH has been reported to improve the enzyme activities and motility [43]. On the other hand, the reduced BWG at higher doses of CSH might be ascribed to increased production of gastric acid, which may lead to gastric ulcers. Our results concur with the research conducted by Ndazigaruye et al. [44], which demonstrated that EAP in low CP diets enhanced the BWG of broilers. The findings are in line with those reported by Maqsood et al. [19], who indicated that EAP improved BWG with a higher lysine ratio in low CP diets. The EAP may facilitate BWG by improving energy digestibility and the availability of AAs in the intestines while reducing their excretion in feces. Moreover, AAs in the free state are digested more rapidly in enterocytes compared to short-chain peptides. Consequently, this augmented BWG may result from the exogenous enzyme-facilitated enhancement of nutrient digestibility and retention [45]. CSH 0.2 + EAP led to a notable enhancement in the FCR of broilers during the entire experiment. These findings align with the research conducted by Nunes et al. [42], which indicated an improvement in the FCR of broilers. The findings are also consistent with the prior studies [19,46], which reported that EAP in low-CP diets optimized FCR in broilers. The growth-enhancing effect of CSH is ascribed to its inhibitory influence on somatostatin and the augmented activity of digestive enzymes [23,47]. Furthermore, animal tissues contain cysteamine that is naturally produced, digested, and quickly eliminated by the body. EAP supplementation may affect the physiological effects of cysteamine; nevertheless, further research is required on this. The enhanced BWG and FCR with protease suggest that exogenous enzyme addition may have augmented the endogenous proteolytic system, enhanced protein digestibility, and optimized intestinal morphology [18]. In the present study, it is revealed that EAP has no impact on the livability of broilers. Our results concur with the research conducted by Maqsood et al. [19], which identified a non-significant interaction between lysine ratio and protease regarding the survivability of broilers. In this experiment, the interaction between CSH and EAP was also determined to be non-significant regarding the survivability of broilers over 35 days. These findings align with the research conducted by Yang et al. [23], which indicated that CSH at a dosage of up to 200 mg/kg has a negligible effect on the survivability of broilers. However, Zavy and Lindsey [41] stated that 1200 mg/kg CSH may cause gastric lesions. Hence, at low doses, CSH does not significantly affect the livability of broilers, which also happened here in this study.

The CSH × EAP interaction was significant for different carcass traits. The CSH 0.2 + EAP therapy exhibited the highest output concerning carcass metrics. These results resemble the study of Ruan et al. [47], who found that CSH increased the longissimus muscle in pigs with a high dressing percentage and lower fat content. Our results also corroborate previous findings [18,19], which described that the addition of EAP improved carcass parameters. The resulting outcomes might be that dietary cysteamine can positively influence intestinal cell morphology, stimulate pancreatic secretions, and improve nutrient digestion. Contrary to this, Nunes et al. [42] concluded that carcass parameters remained unaffected by CSH supplementation. Maqsood et al. [19] also reported that giblet weights were not affected by the addition of protease in the diets of broilers. The enhancement in carcass yield linked to protease treatment may be due to improved protein utilization and deposition [48].

Thiol-containing substances also influence immunity and inflammation by intracellular glutathione and cysteine levels [49]. In the current trial, the CSH × EAP interaction was significant for antibody titers against ND virus and bursa weight. Our findings agreed with Yang et al. [26], who found that CSH addition at the dose rate of 200 mg/kg feed increased the antibody production in the broilers. The CSH supplementation induces the differentiation of IgA-positive cells and intraepithelial lymphocytes in the intestinal mucosa by decreasing the somatostatin-positive cells, thus improving the mucosal immune responses by increasing the levels of IgA [26]. Moreover, CSH inhibits somatostatin in the hypothalamus and increases growth hormone secretion, which enhances B-cell development and antibody production from the immune system [29]. The outcomes are also consistent with Abdollahi et al. [39], who described that EAP increased the immune organs in broilers. An increased weight of immune organs is considered an indicator of immunological advances. This increase in the weight and development of immune organs with CSH and EAP addition may be attributed to enhanced immune cell proliferation and the stimulation of immune-related hormonal activity [50]. Contrary to this, Peek et al. [51] described that EAP had no significant impact on the immune response of broilers. This lack of significant impact on immune response might be due to differences in protease type, enzyme activity level, diet composition, and most importantly health status of birds, as coccidia challenge was induced in birds.

The structure and integrity of the intestinal mucosa are directly related to growth performance. The VL: CD ratio is acknowledged as a crucial measure for intestinal health. Enhanced villous height and deeper crypts result from augmented enterocyte proliferation and villous elongation [52,53,54]. The current investigation revealed a substantial interaction between CSH and EAP for VH and VH: CD in both the duodenum and ileum of broilers; CD remained unaffected by the addition of CSH or EAP. These findings align with the research conducted by Miller et al. [55], which indicated that VH and VH: CD were remarkably increased by CSH in pigs. Similarly, Liu et al. [27] described that VH and VH: CD ratios were increased in broiler chickens with the addition of CSH. The possible reason for this increase in VH is the depletion of somatostatin from the intestinal cells, leading to the increased proliferation and differentiation of intestinal cells [23,27]. Our results corroborate the findings of Maqsood et al. [19], who documented enhanced VH and VH: CD ratios in broilers following the incorporation of EAP. The results revealed that supplementary protease facilitated the digestion of more nutrients [56], and the additional energy absorbed was presumably utilized to enhance gut morphology. The enhanced VL correlates with an elevation in brush border enzymes by improving the digestion and absorption of nutrients [57]. The observed increase in VH and VH: CD ratio indicates improved intestinal health, which has positive implications for nutrient absorption and overall production performance.

The CSH × EAP interaction was significant for nutrient digestion in this study. Our results correspond with the study of Liu et al. [27], who reported that the DM, CP, and OM digestibilities were increased in CSH-supplemented groups. The findings align with the research conducted by Yang et al. [23], which indicated enhanced nutrient digestion because of CSH incorporation in broilers. The enhancements were ascribed to the balanced intestinal microbiota, improved small intestine morphology, and a better mucosal immune system. Alternative explanations for the enhanced nutrient digestibility may stem from the elevated activity of digestive enzymes in the pancreas and intestinal contents resulting from CSH administration [26]. The results reported herein agree with Lu et al. [58], who described that EAP supplementation improved CP and DM absorption. Mahmood et al. [18] also concluded that EAP supplementation improved CP digestibility in broilers. The EAP supplementation increases the retention time and digestion of proteins in the intestine [46], which is also translated into improved overall nutrient utilization efficiency.

## 5. Conclusions

The findings indicate that EAP with CSH in low CP diets positively influences performance, nutrient digestibility, gut histology, immunological response, and carcass characteristics of broilers. The solitary inclusion of each additive provided distinct benefits, such as improved protein digestion, enhanced nutrient absorption, and better physiological responses. However, their combined application yielded synergistic advantages, leading to superior outcomes in key performance indicators, including FCR and gut morphometrics. These findings underscore the potential of integrating CSH at 0.2 g/kg and EAP at 0.2 g/kg into broiler diets as a sustainable strategy to optimize growth and productivity. Further research is warranted to explore the long-term impacts, optimal dosage combinations, and their effects under varying production conditions.

## Figures and Tables

**Table 1:** Experimental layout for trial.

A 2 × 2 Factorial Arrangement Under Completely Randomized Design		Coated Cysteamine Hydrochloride (CSH)	
		CSH 0.2 g/kg	CSH 0.4 g/kg
Exogenous Alkaline Protease (EAP)	No Protease added (−)	Male broilers 150 birds, 6 replicates 25 birds/replicate	Male broilers 150 birds, 6 replicates 25 birds/replicate
	Protease at 0.2 g/kg (+)	Male broilers 150 birds, 6 replicates 25 birds/replicate	Male broilers 150 birds, 6 replicates 25 birds/replicate

CSH 0.2 g/kg = cysteamine hydrochloride at a level of 0.2 g/kg; CSH 0.4 g/kg = cysteamine hydrochloride in diet at 0.4 g/kg; −ve = without protease; +ve = with protease at 0.2 g/kg.

**Table 2:** Feed formulation and nutrient composition of the basal feeds (% as fed basis).

Feed Ingredients (%)	Starter (1–10 d)	Grower (11–24 d)	Finisher (25–35 d)
Corn Grain	54.65	58.54	63.10
Wheat Bran	2.00	1.00	1.27
Rice Polishing	2.80	4.20	5.10
Rice Tips	2.50	3.00	2.00
Molasses	3.00	3.00	3.00
Oil Canola	1.25	1.45	1.70
Soybean Meal	6.50	6.50	6.50
Canola Meal	9.00	8.00	3.20
Sunflower Meal	8.10	5.00	3.80
Fish Meal (52%)	4.00	4.00	4.00
Poultry BP Meal	4.00	4.00	4.00
DL-Methionine	0.13	0.11	0.11
L-Lysine HCl	0.17	0.10	0.12
Limestone	1.80	1.00	1.00
Celite/Marker	-	-	1.00
Micro Mineral Premix *	0.05	0.05	0.05
Vitamin Premix *	0.05	0.05	0.05
**Total**	**100.00**	**100.00**	**100.00**
**Nutrient Contents**			
CP (%)	20.70	19.35	17.55
ME (Calculated, Kcal/kg)	2975	3050	3100
Ca (%)	0.95	0.75	0.65
P (Av) (%)	0.50	0.42	0.36
Lysine (dig) (%)	1.32	1.18	1.08
Methionine (dig) (%)	0.55	0.51	0.48
Arginine (dig) (%)	1.40	1.27	1.17
Tryptophan (dig) (%)	0.21	0.19	0.17
**Analyzed Nutrients**			
CP (%)	20.65	19.28	17.47
Crude fiber (%)	3.72	3.50	3.29
Crude fat (%)	3.50	4.13	4.81
Ca (%)	0.92	0.71	0.63
Ph (Av) (%)	0.51	0.40	0.35
Lysine (dig) (%)	1.30	1.15	1.05
Methionine (dig) (%)	0.52	0.50	0.46
Arginine (dig) (%)	1.39	1.25	1.16
Tryptophan (dig) (%)	0.20	0.17	0.16
Threonine (dig) (%)	0.90	0.80	0.82
Isoleucine (dig) (%)	0.91	0.81	0.73

* The composition of trace mineral and vitamins premix used in the basal diets was the same as previously described by Muneeb et al. [35].

**Table 3:** Effects of CSH with or without the EAP on the performance of broilers.

Items	Treatments				Pooled SEM	*p* Value		
	CSH 0.2 g/kg		CSH 0.4 g/kg					
	−	+	−	+		CSH	Protease	Interaction
**Starter (1–10 d)**								
FI (g)	548	547	547	547	0.81	0.9860	0.7270	0.9785
BWG (g)	293	295	290	291	0.88	0.3392	0.3386	0.2092
FCR	1.05	1.05	1.07	1.07	0.00	0.0672	0.4825	0.1874
**Grower (11–24 d)**								
FI (g)	1457 ^b^	1477 ^a^	1426 ^c^	1458 ^b^	3.90	<0.0001	<0.0001	<0.0001
BWG (g)	979 ^b^	1067 ^a^	920 ^d^	966 ^c^	11.12	<0.0001	<0.0001	<0.0001
FCR	1.34 ^b^	1.23 ^c^	1.41 ^a^	1.35 ^b^	0.01	<0.0001	<0.0001	<0.0001
**Finisher (25–53 d)**								
FI (g)	1103 ^b^	1126 ^a^	1025 ^b^	1103 ^c^	7.96	<0.0001	<0.0001	<0.0001
BWG (g)	910 ^b^	783 ^a^	783 ^d^	871 ^c^	30.03	<0.0001	<0.0001	<0.0001
FCR	1.79 ^c^	1.42 ^d^	1.97 ^a^	1.87 ^b^	0.04	<0.0001	<0.0001	<0.0001
**Overall (1–35 d)**								
FI (g)	3108 ^b^	3150 ^a^	2989 ^c^	3108 ^b^	11.86	<0.0001	<0.0001	<0.0001
BWG (g)	2182 ^b^	2531 ^a^	1992 ^d^	2128 ^c^	41.47	<0.0001	<0.0001	<0.0001
FCR	1.49 ^c^	1.30 ^d^	1.58 ^a^	1.53 ^b^	0.02	<0.0001	<0.0001	<0.0001
Liv %	98.97	99.00	98.31	98.56	0.12	0.0034	0.2288	0.0945
EPEF	442 ^b^	586 ^a^	380 ^d^	419 ^c^	16.30	<0.0001	<0.0001	<0.0001

CSH = cysteamine hydrochloride, Liv = livability, EPEF = European Production Efficiency Factor; CSH 0.2 g/kg = cysteamine hydrochloride at a level of 0.2 g/kg; CSH 0.4 g/kg = cysteamine hydrochloride in diet at 0.4 g/kg; −ve = without protease; +ve = with protease at 0.2 g/kg. Superscripts on different means within row indicate statistical significance at *p* ≤ 0.05.

**Table 4:** Effects of CSH and EAP on the carcass traits in broilers at 35 days (% of live BW).

Items	Treatments				Pooled SEM	*p*-Value		
	CSH 0.2 g/kg		CSH 0.4 g/kg					
	−	+	−	+		CSH	Protease	Interaction
Body weight (g)	2228 ^b^	2567 ^a^	2030 ^d^	2165 ^c^	24.83	<0.0001	<0.0001	<0.0001
Carcass weight (g)	1420 ^b^	1697 ^a^	1240 ^d^	1379 ^c^	20.28	<0.0001	<0.0001	<0.0001
Dressing (%)	63.78 ^b^	66.09 ^a^	61.08 ^c^	63.70 ^b^	0.22	<0.0001	<0.0001	<0.0001
Leg (%)	11.72 ^b^	12.64 ^c^	10.95 ^d^	11.79 ^b^	0.07	<0.0001	<0.0001	<0.0001
Breast (%)	28.35 ^b^	32.16 ^a^	26.42 ^d^	28.08 ^c^	0.25	<0.0001	<0.0001	<0.0001
Heart (%)	0.71 ^a^	0.73 ^a^	0.67 ^b^	0.71 ^a^	0.01	0.0290	0.0178	0.0123
Liver (%)	2.64 ^c^	2.75 ^b^	2.90 ^a^	2.62 ^c^	0.02	<0.0001	<0.0001	<0.0001
Gizzard (%)	1.49	1.57	1.52	1.50	0.02	0.5907	0.3124	0.2268
Fat (%)	2.39 ^d^	2.52 ^b^	2.50 ^c^	2.60 ^a^	0.01	<0.0001	<0.0001	<0.0001

CSH 0.2 g/kg = cysteamine hydrochloride at a level of 0.2 g/kg; CSH 0.4 g/kg = cysteamine hydrochloride in the diet at 0.4 g/kg; −ve = without protease; +ve = with protease at 0.2 g/kg. Superscripts on different means within row indicate statistical significance at *p* ≤ 0.05.

**Table 5:** Effects of CSH and EAP on the immune response of broilers (35 days) (taken as % of live body weight).

Items	Treatments				Pooled SEM	*p*-Value		
	CSH 0.2 g/kg		CSH 0.4 g/kg					
	−	+	−	+		CSH	Protease	Interaction
Bursa (%)	0.37 ^a^	0.39 ^a^	0.30 ^b^	0.36 ^a^	0.01	<0.0001	0.0004	<0.0001
Spleen (%)	0.27	0.30	0.28	0.28	0.01	0.3662	0.3119	0.3596

CSH 0.2 g/kg = cysteamine hydrochloride at a level of 0.2 g/kg; CSH 0.4 g/kg = cysteamine hydrochloride in diet at 0.4 g/kg; −ve = without protease; +ve = with protease at 0.2 g/kg. Superscripts on different means within row indicate statistical significance at *p* ≤ 0.05.

**Table 6:** Effects of CSH and EAP on gut morphometrics of broilers (35 d).

Items		Treatments				Pooled SEM	*p*-Value		
		CSH 0.2 g/kg		CSH 0.4 g/kg					
		−	+	−	+		CSH	Protease	Interaction
Duodenum (µm)	VH	1438 ^b^	1478 ^a^	1412 ^c^	1460 ^ab^	4.81	0.0058	<0.0001	<0.0001
	CD	214	212	216	212	1.67	0.6873	0.3566	0.7814
	VH: CD	6.73 ^ab^	7.02 ^a^	6.57 ^b^	6.91 ^a^	0.05	0.1730	0.0033	0.0150
Ileum (µm)	VH	753 ^bc^	774 ^a^	739 ^c^	765 ^ab^	3.27	0.0491	0.0002	0.0005
	CD	154	155	153	154	0.76	0.5207	0.6833	0.8993
	VH: CD	4.88 ^bc^	4.99 ^a^	4.82 ^c^	4.96 ^ab^	0.02	0.1455	<0.0001	0.0004

CSH 0.2 g/kg = cysteamine hydrochloride at a level of 0. g/kg; CSH 0.4 g/kg = cysteamine hydrochloride diet at 0.4 g/kg; −ve = without protease; +ve = with protease at 0.2 g/kg. Superscripts on different means within row indicate statistical significance at *p* ≤ 0.05.

**Table 7:** Effects of CSH and EAP on nutrient digestibility of broilers at 35 days of age.

Items	Treatments				Pooled SEM	*p*-Value		
	CSH 0.2 g/kg		CSH 0.4 g/kg					
	−	+	−	+		CSH	Protease	Interaction
**Total Tract**								
DM%	71.86 ^b^	72.70 ^a^	69.11 ^d^	71.56 ^c^	0.16	<0.0001	<0.0001	<0.0001
CP%	63.35 ^b^	63.80 ^a^	61.37 ^d^	62.36 ^c^	0.12	<0.0001	<0.0001	<0.0001
OM%	81.39 ^b^	81.57 ^a^	79.11 ^d^	79.92 ^c^	0.12	<0.0001	<0.0001	<0.0001
**Ileum**								
CP%	82.80 ^b^	84.71 ^a^	77.88 ^d^	79.89 ^c^	0.31	<0.0001	<0.0001	<0.0001
OM%	78.42 ^b^	78.92 ^a^	72.56 ^d^	75.46 ^c^	0.30	<0.0001	<0.0001	<0.0001

CSH 0.2 g/kg = cysteamine hydrochloride at a level of 0.2 g/kg; CSH 0.4 g/kg = cysteamine hydrochloride in diet at 0.4 g/kg; −ve = without protease; +ve = with protease at 0.2 g/kg. Superscripts on different means within row indicate statistical significance at *p* ≤ 0.05.

## Data Availability

The raw data supporting the conclusions of this article will be made available by the authors on request.

## List of Abbreviations

AAs: amino acids; AIA: acid-insoluble ash; BWG: body weight gain; CD: crypt depth; CS: cysteamine; CSH: coated cysteamine hydrochloride; DM: dry matter; EAP: exogenous alkaline protease; EPEF: European performance efficiency factors; OM: organic matter; FCR: feed conversion ratio; FI: feed intake; GIT: gastrointestinal tract; HI: hemagglutination inhibition; NDV: Newcastle virus; SBM: soybean meal; VH: villus height; VH: CD: villus height to crypt depth ratio; ME: metabolizable energy; Ca: calcium; P: phosphorous.
